# Side-effects of mdma-assisted psychotherapy: a systematic review and meta-analysis

**DOI:** 10.1038/s41386-024-01865-8

**Published:** 2024-04-23

**Authors:** Julia Colcott, Alexandre A. Guerin, Olivia Carter, Sally Meikle, Gillinder Bedi

**Affiliations:** 1 Melbourne School of Psychological Sciences, University of Melbourne, Melbourne, VIC Australia; 2 Centre for Youth Mental Health, University of Melbourne, and Orygen, Melbourne, VIC Australia

**Keywords:** Outcomes research, Drug development

## Abstract

Evidence suggests that MDMA-assisted psychotherapy (MDMA-AP) has therapeutic potential for treatment of psychiatric illness. We conducted the first comprehensive systematic review and meta-analysis of the side effects of MDMA-AP across indications. We also assessed the quality of side effects-reporting in published trials of MDMA-AP. PubMed, EMBASE, PsycINFO, MEDLINE and Cochrane Central Register of Controlled Trials (CENTRAL) were systematically searched. Phase 2 and 3 MDMA-AP studies were included; Phase 1 studies, which assessed MDMA without psychotherapy, were not. Quality of side effects-reporting was assessed against the CONSORT Harms 2022 guidelines. We also compared numbers of adverse events reported in publications to those recorded in ClinicalTrial.gov registers. Thirteen studies were included, with eight contributing to the meta-analysis. In Phase 2 studies, MDMA-AP was associated with increased odds of any side effect during medication sessions (OR = 1.67, 95%CI (1.12, 2.49)) and in the 7 days following (OR = 1.59, 95%CI (1.12, 2.24)) relative to control conditions. In Phase 3 studies, MDMA-AP was associated with increased odds of any adverse event during the treatment period relative to placebo-assisted psychotherapy (OR = 3.51, 95%CI (2.76, 4.46)). The majority of RCTs were rated as having high risk of bias. Certainty of the evidence was rated as very low to moderate according to the GRADE framework. No included RCT had adequate adherence to the CONSORT Harms 2022 recommendations and reporting rates were also low. Compared to placebo, MDMA-AP was associated with increased odds of side effects, which were largely transient and mild or moderate in severity. However, identified limitations in existing evidence indicate that further investigation is needed to better characterize the safety profile of MDMA-AP and guide implementation.

## Introduction

In the past decade, research on the therapeutic use of 3,4-methylenedioxymethamphetamine (MDMA) in psychotherapy has gathered pace. MDMA-assisted psychotherapy (MDMA-AP) uses a combined pharmacotherapy-psychotherapy model, with MDMA thought to ‘catalyze’ the effects of psychotherapy. In typical protocols, patients attend 3–4 initial ‘preparatory’ psychotherapy sessions, followed by 2–3 full day MDMA- (or placebo-) assisted sessions, each followed by 3–4 ‘integration’ psychotherapy sessions. Methodological questions notwithstanding [1, 2], growing evidence now suggests that MDMA-AP has therapeutic potential for posttraumatic stress disorder (PTSD) [3, 4] and possibly other psychiatric conditions [5–7]. Several clinical trials are planned or ongoing [8].

After a recent decision by the Australian Therapeutic Goods Administration (TGA), psychiatrists who are authorized prescribers can now prescribe MDMA for PTSD [9], making Australia the first country to schedule MDMA for medicinal use. It also appears likely that MDMA-AP will be approved by the U.S. Food and Drug Administration (FDA) for PTSD in 2024 [10], with the first new drug application submitted to the FDA in late 2023. While the literature to date has largely concluded that MDMA [11] and MDMA-AP are safe and well tolerated, concerns exist about the unique potential for substances like MDMA—when combined with psychotherapy—to cause harm [12, 13], as well as inadequacies in the assessment and reporting of adverse events in existing research [14]. A comprehensive understanding of potential safety issues in MDMA-AP is needed to inform both ongoing research and translation to clinical practice.

While acute adverse effects of MDMA without psychotherapy have been determined in detail in placebo-controlled studies in healthy subjects [11], previous reviews of the safety and tolerability of MDMA-AP did not include meta-analyses of safety outcomes [14–22], or were limited to PTSD [15, 16, 20–23], terminal illness [19], or depression [18]. Reviews which included meta-analyses of side effects did not assess the full range of safety outcomes reported [23, 24], and no prior evidence synthesis has included the most recent Phase 3 study of MDMA-AP [4]. Given regulatory changes to the status of MDMA-AP and the large pipeline of ongoing research, a comprehensive updated quantitative assessment of the safety and tolerability of MDMA-AP is timely. Here, we conducted a systematic review and meta-analysis of the side effects of MDMA-AP across psychiatric indications. We also addressed concerns about the adequacy of safety reporting in this research (e.g., [12, 14]), providing the first assessment of the quality of side effect-reporting in published trials of MDMA-AP against the CONSORT Harms 2022 guidelines [25]. Herein, ‘side effects’ is used as an umbrella term to describe the range of safety outcomes reported, which are variously described in published reports as spontaneously reported reactions, treatment emergent adverse events, adverse events, adverse effects and harms.

## Methods

This systematic review and meta-analysis was pre-registered on PROSPERO (CRD42022355572) and is reported according to the PRISMA guidelines [26].

### Search strategy

PubMed, EMBASE, PsycINFO, MEDLINE and Cochrane Central Register of Controlled Trials (CENTRAL) were searched from inception using the following search terms: (“MDMA” OR “3,4-methylenedioxymethamphetamine”) AND (“psychotherapy” OR “therapy”) AND (“safe” OR “side” OR “adverse”), adapted to the technical requirements of each database (Table S1). No restriction on publication date or status was applied.

The primary reviewer (JC) screened titles and abstracts. Full-text articles were reviewed by two independent authors (JC and AAG), with disagreements resolved by consensus. Searches were re-run just before the final analysis on 30 October 2023. Articles were screened and full-texts stored using Covidence (Veritas Health Innovation, Melbourne, Australia).

### Inclusion and exclusion criteria

Studies included were published in English-language peer-reviewed journals and involved administration of one or more MDMA doses to humans (any dose frequency and timing) combined with psychotherapy, with the aim of treating a target psychiatric condition. Exclusion criteria included: (1) studies not including psychotherapy; (2) papers not including original data e.g., reviews or commentaries; (3) conference abstracts; (4) book chapters; and (5) animal studies. These criteria meant that only Phase 2 and 3 studies were included: all Phase 1 studies assessed MDMA in the absence of psychotherapy [11].

### Data extraction

We extracted information on: (1) design; (2) population; (3) demographics (age, sex, ethnicity); (4) MDMA administration (dosage, number of doses); (5) control condition(s); (6) previous MDMA/ecstasy/molly use; (7) health screening; (8) medical comorbidity; (9) concomitant medications; (10) timing of side effects assessment; (11) outcomes of interest (i.e., side effects, study withdrawal); and (12) whether structured assessments were used to assess side effects or safety.

Data were extracted by the primary reviewer (JC), and subsequently checked for accuracy and completeness by a second reviewer (AAG or SM). For missing data, reviewers first contacted study authors for unreported data and/or additional details. If additional information was not available, missing data were coded as “Not Reported”.

### Data synthesis

Qualitative synthesis included the following steps: (1) data were extracted; (2) quality and risk of bias was assessed; and (3) findings were summarized in a table for each outcome. Results were synthesized using a narrative approach.

Where at least two studies contributed data for an outcome, we conducted pairwise meta-analyses using Review Manager 5.4 (The Cochrane Collaboration, United Kingdom), comparing MDMA-AP to the control. Various doses for the MDMA group were pooled, and control groups included both active (low-dose MDMA) and inactive placebo. As outcomes were dichotomous, odds ratios (ORs) with 95% confidence intervals (95% CI) were calculated (Mantel–Haenzsel method). Random-effects models were used to control for heterogeneity. The significance level was set to *p* < 0.05 (two-tailed). Publication bias was not assessed as there were ≤10 studies in each analysis [27]. Heterogeneity was estimated using the I² statistic, and subgroup analyses of PTSD studies and non-PTSD studies were completed (it was not possible to conduct more specific subgroup analyses as to particular types of PTSD patients—e.g., combat veterans – as most studies included participants with a range of different types of trauma exposure). Sensitivity analyses were performed to assess effects of method used to assess side effects and number of medication sessions on the outcomes.

We synthesized outcomes from Phase 2 and 3 studies separately due to differences in data collection and reporting. We defined the following primary outcomes for Phase 2 studies: (1) any side effect—during medication sessions; (2) any side effect—7 days following medication sessions; (3) treatment emergent adverse event (TEAE); and (4) psychiatric treatment emergent adverse event. Outcomes 1 and 2 were based on ‘spontaneous reported reactions’ data from Phase 2 studies, which included a subset of events that were judged to be expected based on evidence about MDMA’s side effects from healthy control studies [11]. TEAEs and psychiatric TEAEs were defined as events not on the expected reactions list, or which continued for >7 days after medication sessions [28]. We defined the following primary outcomes for Phase 3 studies: (1) treatment emergent adverse event; and (2) adverse event of special interest. Consistent with Phase 3 study [3, 4] reporting, we defined TEAEs for Phase 3 outcomes as any adverse event occurring across the treatment period. Adverse events of special interest were prespecified events related to cardiac function, suicidality, and MDMA abuse, misuse, or diversion identified as of special interest by the FDA [3, 4]. We also defined study withdrawal as a primary outcome across all studies. Secondary outcomes broke down each primary outcome by type (e.g., anxiety, reason for withdrawal; see Supplemental Table 8 for primary and secondary outcomes).

### Quality of side effect-reporting

Data on side effects from all included randomized controlled trials (RCTs) were independently assessed by two reviewers (JC and AAG) using the CONSORT Harms 2022 guideline, a 17-item checklist for reporting of harms in randomized trials [25]. Each checklist item was scored individually (1 = adequately reported; 0 = inadequately or not reported at all) (e.g., [29, 30]). Overall adherence to the CONSORT Harms guideline was calculated by dividing the sum of individual scores by the total number of items to generate a percentage. Adherence of ≥70% was defined as adequate [30].

The 2007 FDA Amendments Act mandated the reporting of all clinical trial results, including adverse events, in the ClinicalTrials.gov database [31]. To assess whether the adverse events reported in published articles aligned with those recorded in ClinicalTrial.gov, we compared the total number of serious and ‘non-serious’ adverse events from each of these sources for each trial.

### Quality and risk of bias assessment

The outcome being assessed for risk of bias was side effects. For the extracted RCTs, two reviewers (JC and AAG) independently assessed bias using the Cochrane Risk of Bias Tool for randomized trials (RoB 2 [32]), which evaluates bias arising from the randomization process, deviations from intended interventions, missing outcome data, outcome measurement and selection of the reported result. Cohen’s kappa was calculated for overall bias to determine agreement between reviewers. Any conflict between reviewers was resolved through discussion to reach consensus.

Further, based on the approach of the Cochrane Adverse Effects Methods Group [33] consideration was given to the following issues that affect the quality of side effects-data: (1) how side effects data were collected (spontaneous reporting, use of structured scales or questionnaires); (2) how side effects were reported (systematically or ad hoc); and (3) the presence of a control group.

The GRADE framework was used to assess the overall certainty of evidence for each outcome [34]. Under the GRADE approach, RCT evidence is initially graded as high but then downgraded to lower levels depending on risk of bias, inconsistency, indirectness, imprecision and publication bias. Two reviewers (JC and AAG) completed this analysis, categorizing each outcome as having very low, low, moderate or high certainty.

## Results

### Study selection

**Fig. 1:**
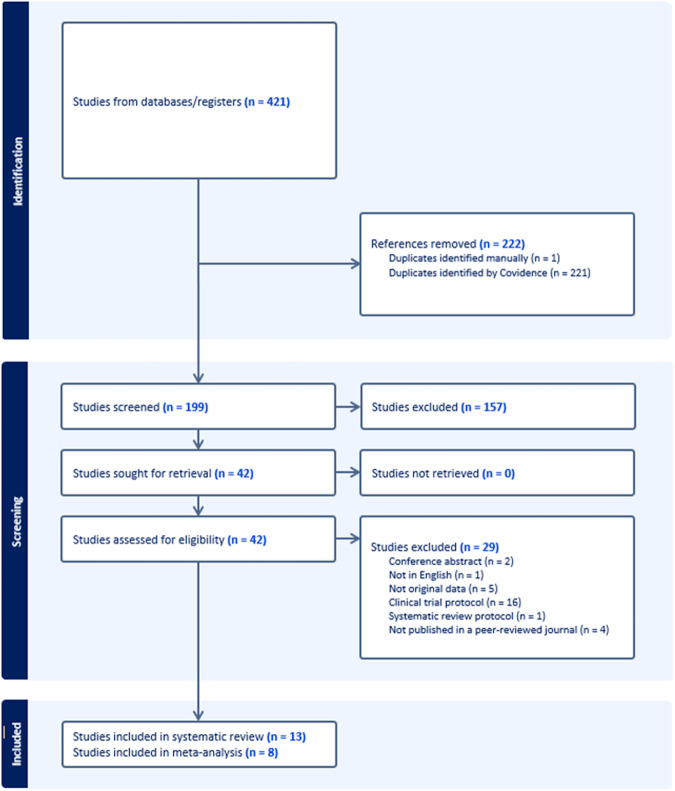
PRISMA flow diagram. Flowchart describing the search strategy, including identification, screening and inclusion of studies.

Thirteen studies met inclusion criteria for the systematic review, involving 333 unique participants (Fig. 1). Characteristics of eligible studies are in Table 1. Five studies were excluded from meta-analyses because there was no control group (Table S2). The remaining eight RCTs included 298 participants, and reported 138 different safety outcomes, of which 64 provided sufficient data for meta-analysis (i.e., at least two studies per outcome; Table S3). A description of which studies contributed data to each analysis is provided in Table S4.

**Table 1:** Characteristics of included studies.

Study	Population/Sample characteristics	MDMA condition	Control condition	Sample MDMA/ ecstasy/molly use (%)	Side effects assessment	Reporting of side effects	Side effects in MDMA group [number of participants (% of participants) unless otherwise specified]
Bouso 2008, RCT, Spain	PTSD *N* = 6 Age (SD) = 35.7 (7.3) Sex (% female) = 100 Ethnicity not reported	Single medication session Group 1 (*n* = 3): 50 mg Group 2 (*n* = 1): 75 mg	Inactive placebo (*n* = 2)	Baseline: 0%	UKU Scale of Secondary Effects—24 h and 5–7 days after medication session Blood pressure and heart rate - every 30 min for 6 h during medication session	Only reported for 2 subjects, 24 h after medication session	**24 h after MDMA session (50 mg + 75 mg):** Asthenia/Lassitude/ Increased Fatigability = 2 (50%) Sleepiness/Sedation = 2 (50%) Tension/Inner Unrest = 2 (50%) Tension headache = 2 (50%) **24 h after MDMA session (50 mg):** Asthenia/Lassitude/ Increased Fatigability = 1 (33%) Sleepiness/Sedation = 1 (33%) Tension/Inner Unrest = 1 (33%) Tension headache = 1 (33%) **24 h after MDMA session (75 mg):** Asthenia/Lassitude/ Increased Fatigability = 1 (100%) Sleepiness/Sedation = 1 (100%) Tension/Inner Unrest = 1 (100%) Tension headache = 1 (100%)
Danforth 2018, RCT, USA	Autistic adults with social anxiety *N* = 12 Age (SD) = 31.3 (8.8) Sex (% female) = 16.7 Ethnicity (% Caucasian) = 50	Two medication sessions Group 1 (*n* = 4): 75 mg first session, 100 mg second session Group 2 (*n* = 4): 100 mg first session, 125 mg second session	Inactive placebo (*n* = 4)	Baseline: 0%	Spontaneously reported reactions - during medication sessions and 7 days after TEAEs - after 1st medication session until primary end point; primary end point to 6-month follow-up C-SSRS - Beginning and end of medication sessions SUDs - Hourly during medication sessions Blood pressure, heart rate and temperature - Pre-medication, hourly for 6–7 h during medication sessions	All data reported, separated by group All data reported, by SOC, separated by group Only psychiatric TEAEs broken down by type	**During MDMA sessions:** Anxiety = 6 (75%) Difficulty concentrating = 5 (63%) Fatigue = 4 (50%) Headache = 4 (50%) Sensitivity to cold = 4 (50%) **7 days following MDMA sessions:** Fatigue = 5 (63%) Headache = 5 (63%) Difficulty concentrating = 4 (50%) Lack of appetite = 3 (38%) Need more sleep = 3 (38%) **Psychiatric TEAEs**: Depressed mood = 2 (25%) Suicidal ideation = 2 (25%) Anxiety = 1 (13%) Depression = 1 (13%) Panic attack = 1 (13%) Panic reaction = 1 (13%) TEAEs 6-month follow-up Psychiatric Disorders = 4 (50%)
Jardim 2021, Open-label, Brazil	PTSD *n* = 3 Age (SD) = 40.3 (5.0) Sex (% female) = 66.7 Ethnicity (% Caucasian) = 66.7	Three medication sessions 75 mg first session, 75 or 125 mg second and third sessions (*n* = 3) Supplemental half-dose after 1.5–2 h (optional)	N/A	Not reported	Spontaneously reported adverse events - Preparatory, MDMA, integrative sessions, and 7 days after MDMA sessions C-SSRS, DES-II - Baseline and two months after the final MDMA session SUDs - Hourly during MDMA sessions Blood pressure, heart rate and temperature - During MDMA sessions (measurement intervals not reported)	All data reported, by number of events	**Reported by number of events (% of sessions)** **During MDMA sessions**: Somatic pains = 4 (44%) Cough = 3 (33%) Anguish = 2 (22%) Colic = 2 (22%) **7 days following MDMA sessions**: Anguish = 10 (14%) Headache = 3 (4%) Somatic pains = 3 (4%) Fatigue = 3 (4%) **Other integrative sessions**: Somatic pains = 8 (44%) Anguish = 6 (33%) Cough = 3 (17%) Fear = 3 (17%)
Jerome 2020, LTFU, pooled analysis of 6 phase 2 trials (including Mithoefer 2011, 2018, Ot’alora 2018, Oehen 2013), USA, Canada, Switzerland, Israel	PTSD *N* = 107^a^ Age (SD) = 40.5 (10.6) Sex (% female) = 57.9 Ethnicity (% Caucasian) = 89.7	Two to three medication sessions 75–125 mg (*n* = 74)	*n* = 31 Inactive or active placebo (25–40 mg MDMA)	Baseline: 29.9% LTFU: 9.6% used ecstasy/ MDMA after treatment exit and LTFU	LTFUQ - 12 months post-treatment	All data reported, but not for MDMA and control arms separately, due to open-label cross-over designs	**LTFU - 12 months (both MDMA and control arms)**: Other harms = 3 (5%) Worsened mood = 3 (4%) Increased nightmares, flashbacks, or intrusive memories = 2 (2%) Difficulty feeling emotions = 2 (2%) Avoiding people or places = 2 (2%) Increased anxiety = 2 (2%) Excessive vigilance = 2 (2%) Less involved in the community/world around me = 2 (2%)
Mitchell 2021, RCT, USA, Canada, Israel	PTSD *N* = 90 Age (SD) = 41.0 (11.9) Sex (% female) = 65.6 Ethnicity (% Not Hispanic or Latino) = 90	Three medication sessions 80 mg first session, 120 mg second and third sessions (*n* = 46) Supplemental half-dose after 1.5–2.5 h (optional)	Inactive placebo (*n* = 44)	Baseline: Lifetime: 32.2% Past 10 years: 21.1%	Spontaneously reported TEAEs - From first medication session to last integration session Monitoring of AESIs - cardiac function, suicidal ideation, and abuse potential for MDMA C-SSRS - Each study visit Blood pressure, body temperature and heart rate - Pre-medication, before supplemental dose, end of medication session	Only the most common ( > 5% of subjects) TEAEs related to MDMA reported All data reported, separated by group	**TEAEs**: Muscle tightness = 29 (63%) Decreased appetite = 24 (52%) Nausea = 14 (30%) Hyperhidrosis = 9 (20%) Feeling cold = 9 (20%) **AESIs**: Suicidal ideation = 2 (4%) Intentional self-harm in the context of suicidal ideation = 1 (2%)
Mitchell 2023, RCT, USA, Israel	PTSD *N* = 104 Age (SD): MDMA = 38.2 (11.0); Placebo = 40.0 (9.6) Sex (% female): MDMA = 60.4; Placebo = 82.4 Ethnicity (% Not Hispanic or Latino): MDMA = 67.9; Placebo = 76.5	Three medication sessions 80 mg first session, 120 mg second and third sessions (*n* = 53) Supplemental half-dose after 1.5–2 h (optional)	Inactive placebo (n = 51)	Baseline: Lifetime: MDMA = 41.5%; Placebo = 51.0% Past 10 years: MDMA = 24.5%; Placebo = 35.3%	Spontaneously reported TEAEs - From first medication session to last integration session Monitoring of AESIs - cardiac function, suicidal ideation, and abuse potential for MDMA C-SSRS - Each study visit Blood pressure, body temperature and pulse - Pre-medication, ~2 h post-initial dose, end of medication session	Only the most common (>10% of subjects) TEAEs related to MDMA reported Most common (>5% of subjects) psychiatric TEAEs reported All cardiac/vascular TEAEs reported All severe psychiatric TEAEs reported All data reported, separated by group	**TEAEs**: Muscle tightness = 31 (58.5%) Nausea = 24 (45.3%) Decreased appetite = 19 (35.8%) Hyperhidrosis = 18 (34.0%) Feeling hot = 14 (26.4%) **Psychiatric TEAEs**: Suicidal ideation = 18 (34.0%) Insomnia = 19 (35.8%) Anxiety = 15 (28.3%) Restlessness = 8 (15.1%) Bruxism = 7 (13.2%) **Cardiac/vascular TEAEs**: Palpitations = 5 (9.4%) Flushing = 3 (5.7%) Tachycardia = 2 (3.8%) Peripheral coldness = 2 (3.8%) **AESIs**: Suicidal ideation = 2 (3.8%) Non-suicidal self-injurious behavior = 1 (1.9%) Palpitations = 4 (7.5%)
Mithoefer 2018, RCT, open-label cross-over, USA	PTSD *N* = 26 Age (SD) = 37.2 (10.3) Sex (% female) = 27 Ethnicity (% Caucasian) = 85	Two medication sessions Group 1 (*n* = 7): 75 mg Group 2 (*n* = 12): 125 mg Open-label arm: Group 1 (*n* = 6): Two additional medication sessions, 100–125 mg Group 2 (*n* = 12): One additional medication session, 125 mg Supplemental half-dose after 1.5–2 h (optional)	Active placebo: 30 mg MDMA (*n* = 7) Open-label arm (*n* = 6): Three medication sessions, 125 mg Supplemental half-dose after 1.5–2 h (optional)	Baseline: 23%	Spontaneously reported reactions—during medication sessions and 7 days after TEAEs - until 2 months following the last medication session if requiring medical attention C-SSRS - at all visits and twice during the 7 days after medication sessions Blood pressure and heart rate - Every 15 min for the first 4 h, then every 30 min. Body temperature - 60–90 min intervals	Only most common spontaneously reported reactions reported (threshold not reported) All data reported, by SOC, separated by group Only psychiatric TEAEs broken down by type	**During MDMA sessions (75 + 125 mg)**: Anxiety = 17 (89%) Fatigue = 11 (58%) Headache = 13 (68%) Jaw clenching or tight jaw = 13 (68%) **During MDMA sessions (75 mg)**: Anxiety = 6 (86%) Fatigue = 4 (57%) Headache = 5 (71%) Jaw clenching or tight jaw = 4 (57%) **During MDMA sessions (125 mg)**: Anxiety = 11 (92%) Fatigue = 7 (58%) Headache = 8 (67%) Jaw clenching or tight jaw = 9 (75%) **7 days following MDMA sessions (75 + 125 mg)**: Fatigue = 17 (89%) Anxiety = 15 (79%) Need more sleep = 15 (79%) Insomnia = 13 (68%) **7 days following MDMA sessions (75 mg)**: Fatigue = 7 (100%) Anxiety = 5 (71%) Need more sleep = 6 (86%) Insomnia = 3 (43%) **7 days following MDMA sessions (125 mg)**: Fatigue = 10 (83%) Anxiety = 10 (83%) Need more sleep = 9 (75%) Insomnia = 10 (86%) **Psychiatric TEAEs (75 + 125 mg)**: Anxiety = 1 (5%) Flashbacks = 1 (5%) Tic = 1 (5%) **Psychiatric TEAEs (75 mg)**: Anxiety = 0 (0%) Flashbacks = 0 (0%) Tic = 0 (0%) **Psychiatric TEAEs (125 mg)**: Anxiety = 1 (8%) Flashbacks = 1 (8%) Tic = 1 (8%)
Mithoefer 2011, RCT, open-label cross-over, USA	PTSD *N* = 20 Age (SD) = 40.4 (7.2) Sex (% female) = 85 Ethnicity (% Caucasian) = 100	Two medication sessions 125 mg (*n* = 12) Open-label arm (*n* = 5): One medication session, 125 mg Supplemental half-dose after 2–2.5 h (optional)	Inactive placebo (n = 8) Open-label arm: Two medication sessions, 125 mg (n = 7) One additional medication session, 125 mg (n = 4) Supplemental half-dose after 2–2.5 h (optional)	Baseline: 45%	Spontaneously reported reactions - during medication sessions and 7 days after RBANS; PASAT; RCFT - Baseline and 2 months after second medication session Blood pressure, pulse, and temperature - During medication sessions (measurement intervals not reported)	All data reported by number of events, separated by group No reporting of TEAEs	**Reported by number of events (% of sessions)** **During MDMA sessions:** Anxiety = 14 (58%) Headache = 14 (58%) Insomnia = 13 (54%) Nausea = 12 (50%) **7 days following MDMA sessions:** Tight jaw = 19 (79%) Fatigue = 18 (75%) Anxiety = 13 (54%) Low mood = 10 (42%)
Monson 2020, Open-label, USA	Couples, one diagnosed with PTSD *N* = 12 (six couples) Age (SD): Patient = 47.1 (12.5); Partner = 46.6 (11.2) Sex (% female): Patient = 40; Partner = 66.7 Ethnicity (% Caucasian): Patient = 100; Partner = 100	Two medication sessions for each partner (*n* = 12), 75 mg first session, 100 mg second session Supplemental half-dose after 1.5 h (optional)	N/A	Not reported	Spontaneously reported reactions - during medication sessions and 7 days after Adverse events - from enrolment to study completion and 3 and 6-month follow-up	All data reported All data reported, by SOC	**During MDMA sessions:** Anxiety = 9 (75%) Headache = 8 (67%) Lack of appetite = 10 (83%) Jaw tightness = 7 (58%) **7 days following MDMA sessions, reported by number of events (% of sessions):** Anxiety = 29 (35%) Fatigue = 22 (26%) Headache = 19 (23%) Insomnia = 18 (21%) Lack of appetite = 18 (21%) **TEAEs:** Upper respiratory tract infection = 3 (25%) Anxiety = 2 (17%) Disturbance in attention = 2 (17%) Fatigue = 2 (17%) Decreased appetite = 2 (17%) Nausea = 2 (17%) **TEAEs 6-month follow-up:** Anxiety = 1 (8%)
Oehen 2013, RCT, open-label cross-over, Switzerland	PTSD *N* = 12 Age (SD) = 41.4 (11.2) Sex (% female) = 83 Ethnicity not reported	*n* = 8 Three medication sessions 125 mg Open-label arm (*n* = 3): Two medication sessions, 150 mg Supplemental half-dose after 2.5 h (optional)	n = 4 Active placebo, 25 mg MDMA Open-label arm (*n* = 4): Three medication sessions, 125 mg Supplemental half-dose after 2.5 h (optional)	Baseline: 8.3%	Spontaneously reported reactions - during medication sessions and 7 days after SUDS - During medication sessions (measurement intervals not reported) Blood pressure and heart rate - 15 and 5 min before MDMA administration, every half-hour for 4 h then every hour Body temperature - 15 min before MDMA administration then hourly	Reported aggregately across randomized and open-label arms, by number of events No reporting of TEAEs	**Reported by number of events (% of sessions)** **During MDMA sessions (125 + 150 mg):** Insomnia = 19 (44%) Jaw clenching = 18 (42%) Lack of appetite = 17 (40%) Impaired gait/balance = 16 (37%) **During MDMA sessions (125 mg):** Insomnia = 16 (43%) Jaw clenching = 16 (38%) Lack of appetite = 15 (41%) Impaired gait/balance = 12 (32%) **During MDMA sessions (150 mg):** Insomnia = 3 (50%) Jaw clenching = 4 (67%) Lack of appetite = 2 (33%) Impaired gait/balance = 4 (67%) **7 days following MDMA sessions (125 + 150 mg):** Anxiety = 11 (26%) Fatigue = 24 (56%) Insomnia = 20 (47%) Low mood = 20 (47%)
Oehen & Gasser 2022, Case series, Switzerland	Various treatment-resistant disorders *N* = 50 MDMA only (*n* = 6) MDMA and LSD (*n* = 18) Age: Median Clinician 1: 48.5 Clinician 2: 45 Total mean age not reported Sex (% female) = 34 Ethnicity not reported	Group and individual sessions, 75–125 mg “Rare” cases: supplemental dose of 50 mg after ~2 h	N/A	Previous recreational use of psychedelics (<4 occasions): 24% Not reported for MDMA individually	Clinical observation	Descriptive, ad-hoc reporting	**Reported descriptively:** Patients sitting up, walking around, obsessively taking notes Chattering Intense emotions Overwhelm Distress Suicidal thoughts Mild self-harm **Reasons for termination of treatment:** Compliance problems Difficult first psychedelic experiences Unresolved transference conflict with therapist
Ot’alora 2018, RCT, open-label cross-over, USA	PTSD *N* = 28 Age (SD) = 42.0 (12.9) Sex (% female) = 67.9 Ethnicity (% Caucasian) = 92.9	Two medication sessions Group 1 (*n* = 9): 100 mg Group 2 (*n* = 13): 125 mg Open-label arm (*n* = 21): One session, 100–125 mg Supplemental half-dose after 1.5 h (optional)	Active placebo (*n* = 6): 40 mg MDMA Open-label arm (n = 5): Three medication sessions 100–125 mg MDMA Supplemental half-dose after 1.5 h (optional)	Not reported	Spontaneously reported reactions - during medication sessions and 7 days after TEAEs - until two months following the last open-label session. SAEs and TEAEs that represented a change in psychiatric status recorded until 12-month follow-up C-SSRS - each visit and on two of seven days following medication sessions Blood pressure and heart rate - before medication administration, every half-hour for the first four hours, then hourly until six hours after ingestion Temperature - Hourly during medication sessions	Only most common spontaneously reported reactions reported (⩾40% of participants in any group) All data reported, by SOC, separated by group Only psychiatric TEAEs broken down by type	**During MDMA sessions (100 + 125 mg):** Anxiety = 13 (59%) Jaw clenching, tight jaw = 13 (59%) Muscle tension = 11 (50%) Dizziness = 9 (41%) **During MDMA sessions (100 mg):** Anxiety = 6 (67%) Jaw clenching, tight jaw = 5 (56%) Muscle tension = 4 (44%) Dizziness = 2 (22%) **During MDMA sessions (125 mg):** Anxiety = 7 (54%) Jaw clenching, tight jaw = 8 (62%) Muscle tension = 7 (54%) Dizziness = 7 (54%) **7 days following MDMA sessions (100 + 125 mg):** Anxiety = 18 (82%) Fatigue = 16 (73%) Low mood = 15 (68%) Insomnia = 13 (59%) Need more sleep = 13 (59%) **7 days following MDMA sessions (100 mg):** Anxiety = 8 (89%) Fatigue = 7 (78%) Low mood = 6 (67%) Insomnia = 7 (78%) Need more sleep = 5 (56%) **7 days following MDMA sessions (125 mg):** Anxiety = 10 (78%) Fatigue = 9 (69%) Low mood = 9 (69%) Insomnia = 6 (46%) Need more sleep = 8 (62%) **Psychiatric TEAEs (100 + 125 mg):** Anxiety = 7 (32%) Depressed mood = 4 (18%) Irritability = 3 (14%) Obsessive rumination = 2 (9%) **Psychiatric TEAEs (100 mg):** Anxiety = 3 (33%) Depressed mood = 2 (22%) Irritability = 2 (22%) Obsessive rumination = 1 (11%) **Psychiatric TEAEs (125 mg):** Anxiety = 4 (31%) Depressed mood = 2 (15%) Irritability = 1 (8%) Obsessive rumination = 1 (8%)
Sessa 2021, Open-label, UK	Alcohol Use Disorder, *N* = 14 Age = 48 Sex (% female) = 42.9 Ethnicity not reported	Two medication sessions, 125 mg Supplemental half-dose after 2 h (optional)	N/A	Not reported	Spontaneously reported adverse events SUDS - Hourly during MDMA sessions Temperature, blood pressure and heart rate - t = 0, before MDMA administration, half-hourly up to t = 2 h, then hourly for a minimum of six hours after ingestion C-SSRS - Screening, baseline, throughout the eight-week therapy course, in the week after each MDMA session and at three, six and nine-month follow-up.	Not reported	Not reported

*AESI* Adverse Event of Special Interest, *C-SSRS* Columbia Suicide Severity Rating Scale, *CAPS* Clinician-Administered PTSD Scale, *DES-II* Dissociative Experience Scale-II, *LTFU* Long-Term Follow-up, *PASAT* The Paced Auditory Serial Addition Task, *RBANS* Repeatable Battery for the Assessment of Neuropsychological Status, *RCFT* Rey-Osterrieth Complex Figure, *RCT* randomized controlled trial, *SAE* Serious adverse event, *SOC* system organ class, *SUDs* subjective units of distress, *TEAE* treatment emergent adverse event.

Spontaneously reported reactions: subset of adverse events that could be expected based on findings from published studies in healthy volunteers.

^a^Eight participants did not complete treatment, and six of the eight participants underwent at least one medication session prior to discontinuing study participation.

### Systematic review: measurement of side effects

Of 13 studies included in the systematic review, most (*n* = 11) used passive monitoring of side effects [3–6, 35–41]. One [42] systematically assessed side effects using the UKU Scale of Secondary Effects [43], while another formally assessed long-term follow-up outcomes, including harms, using a Long-Term Follow-Up Questionnaire [44]. Use of scales to systematically assess specific side effects was mixed. Eight studies assessed suicidal ideation and behavior with the Columbia Suicide Severity Rating Scale [3–6, 35, 36, 40, 41]. In addition, one study [36] measured cognitive function using the Paced Auditory Serial Addition Task [45], Repeatable Battery for the Assessment of Neuropsychological Status [46] and Rey-Osterrieth Complex Figure [47], while another [35] assessed dissociation using the Dissociative Experience Scale-II [48]. Most studies measured vital signs at regular intervals during medication sessions [3–6, 35, 36, 38, 40–42]. Four also assessed subjective units of distress (SUDs) during medication sessions [5, 6, 35, 38].

### Systematic review: reporting of side effects

Phase 2 and 3 studies used different methods to report side effects. Phase 2 studies [6, 36–38, 40, 41] typically reported ‘spontaneously reported reactions’ – a subset of adverse events that could be expected based on findings from healthy volunteer studies [11] – during and 7 days following medication sessions [28]. These were collected and reported separately to TEAEs, defined as events not on the expected reactions list, or which continued for >7 days after medication sessions [28]. In contrast, Phase 3 studies [3, 4] did not have separate data collection for spontaneously reported reactions and TEAEs, instead only reporting TEAEs across the entire treatment period. In addition, Phase 3 studies monitored and reported Adverse Events of Special Interest (AESI) relating to cardiac function, suicide risk and MDMA abuse, misuse or diversion. AESIs were prespecified based on FDA guidance [3, 4]. Only four studies reported sides effects by dose administered [38, 40–42], while all other studies either combined doses in their analyses, or only use one dose.

### Meta-analysis

**Fig. 2:**
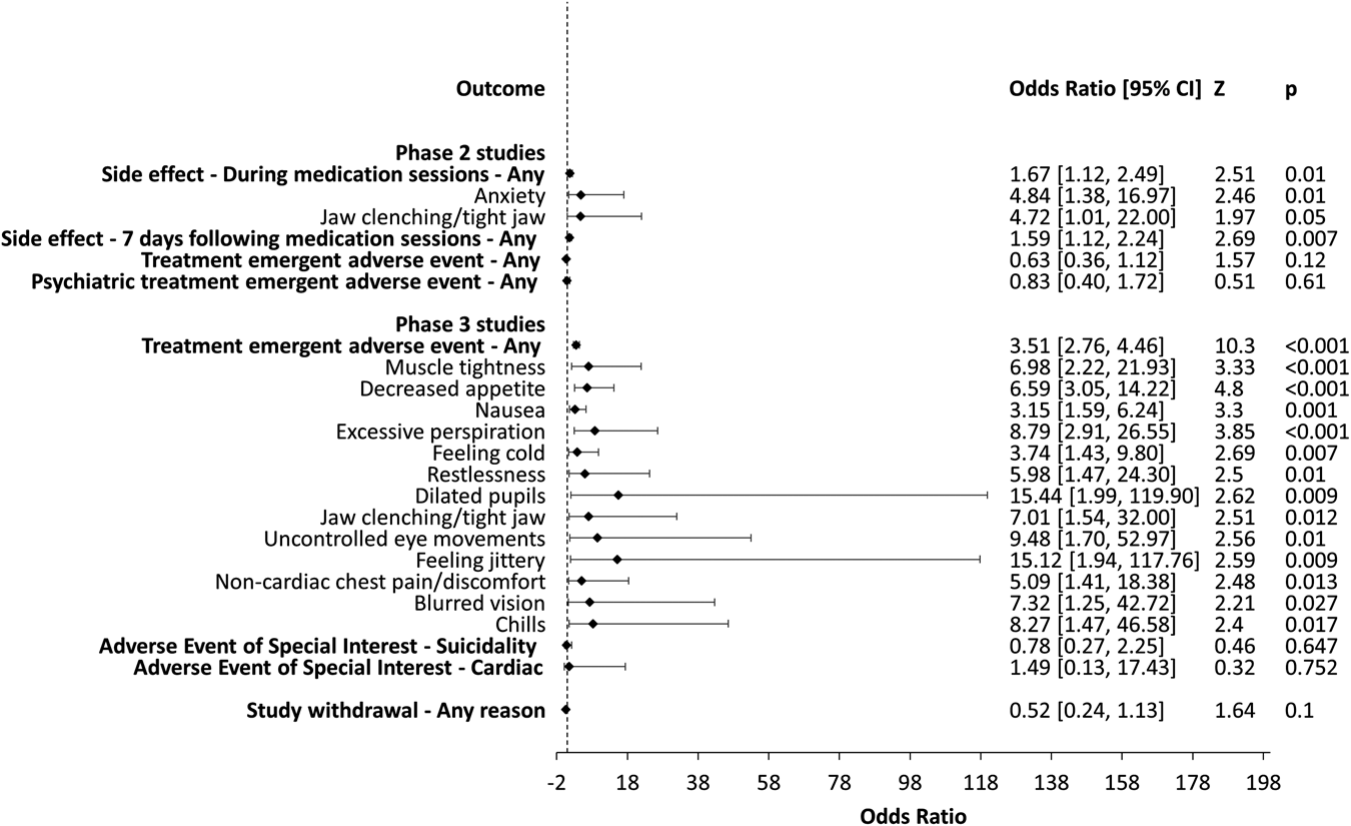
Summary of meta-analysis results. Odds ratios (OR) and 95% confidence intervals (CIs) comparing MDMA-AP with control groups on all primary outcomes and secondary outcomes that achieved statistical significance (*p* < 0.05). OR > 1 indicates an increased likelihood of the event when treated with MDMA-AP compared with control groups and an OR < 1 indicates a reduced likelihood. Phase 2 studies: odds of experiencing any side effects during medication sessions was higher in the MDMA-AP group compared to control groups (*p* = 0.01), with anxiety and jaw-clenching more likely in the MDMA-AP group compared to control groups (*ps* < 0.05). Odds of experiencing any side effect in the 7 days following medication sessions were also higher in the MDMA-AP group compared to control groups (*p* = 0.007). Phase 3 studies: odds of experiencing any treatment emergent adverse events was higher in the MDMA-AP group compared to control groups (*p* < 0.001), with muscle tightness, decreased appetite, nausea, excessive perspiration, feeling cold, restlessness, dilated pupils, jaw clenching/tight jaw, uncontrolled eye movements, feeling jittery, non-cardiac chest pain/discomfort, blurred vision, and chills more likely in the MDMA-AP group compared to control groups (*ps* < 0.05). There were no other significant associations in Phase 2 or 3 studies.

We report on primary outcome measures below, together with any secondary outcomes achieving statistical significance (*p* < 0.05; Fig. 2). Sub-group analyses of PTSD studies are provided in Fig. S1. Other secondary outcomes are presented in Table S3.

#### Phase two studies

##### Side effects—during medication sessions

In Phase 2 studies, the odds of experiencing any side effect during medication sessions were higher in the MDMA-AP group compared with controls. In total, 45% of MDMA-AP participants versus 30% of controls reported side effects during medication sessions. Odds of experiencing anxiety and jaw clenching during medication sessions were also higher in MDMA-AP than control participants. Based on dose-dependent reports [38, 41], jaw clenching may be more likely to occur when receiving a higher dose (125 or 150 mg). All other specific side effects during medication sessions did not reach significance (Table S3). In PTSD studies only, anxiety was also more common under MDMA-AP than controls. All other results for PSTD studies did not reach significance.

##### Side effects—7 days following medication sessions

In Phase 2 studies, the odds of experiencing any side effect in the 7 days following medication sessions were higher in the MDMA-AP group compared with controls. In total, 46% of MDMA-AP participants versus 31% of controls reported side effects in the 7 days following medication sessions. No specific side effects in the 7 days following medication sessions reached significance (Table S3). In PTSD studies only, the odds of experiencing any side effect in the 7 days following medication sessions were higher in the MDMA-AP group compared with controls, as were the odds of experiencing anxiety during this time. No other results for PSTD studies reached significance.

##### Treatment emergent adverse events (TEAE)

In Phase 2 studies, there was no difference in the odds of experiencing any TEAE in the MDMA-AP group compared with controls, and all specific TEAEs were also non-significant (Table S3). In PTSD studies only, the odds of experiencing any TEAE were lower in the MDMA-AP group compared with control. All other results for PSTD studies did not reach significance.

There was no difference in the odds of experiencing any psychiatric TEAE in the MDMA-AP group compared with controls, and all specific psychiatric TEAEs were also non-significant (Table S3). The same results were observed in PSTD studies only.

#### Phase three studies

##### Treatment emergent adverse events (TEAE)

In Phase 3 studies, the odds of experiencing any TEAE in the MDMA-AP group compared with controls was higher, with 16% of MDMA-treated participants reporting TEAEs, compared with 5% of those treated with placebo. MDMA-AP was associated with increased odds of muscle tightness; decreased appetite; nausea; excessive perspiration; feeling cold; restlessness; dilated pupils; jaw clenching/tight jaw; uncontrolled eye movements; feeling jittery; non-cardiac chest pain/discomfort; blurred vision; and chills. All other specific TEAEs were non-significant (Table S3). Both Phase 3 studies focused on PSTD, so no sub-group analyses were performed.

##### Adverse event of special interest (AESI)

In Phase 3 studies, there was no difference in the odds of experiencing an AESI related to suicidality in the MDMA-AP group compared with controls, and all specific AESIs related to suicidality were also non-significant (Table S3). Similarly, there was no difference in the odds of experiencing an AESI related to cardiac function in the MDMA-AP group compared with controls, and all specific AESIs related to cardiac function were also non-significant (Table S3). No AESIs related to MDMA abuse, misuse or diversion were reported. As above, no sub-group analyses were performed.

#### Phase 2 and 3 studies

##### Withdrawal

Across all studies, there was no difference in the odds of withdrawing from the study for any reason in the MDMA-AP group compared with controls, and all specific reasons for withdrawal were also non-significant (Table S3). In PTSD studies only, all results were also non-significant.

#### Sensitivity analyses

Sensitivity analyses assessing effects of method used to assess side effects and number of medication sessions did not change outcomes.

### Risk of bias

**Fig. 3:**
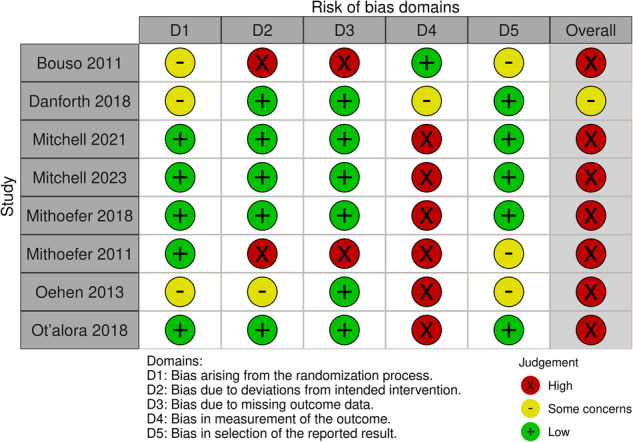
Quality assessment of studies included in the meta-analyses. Overall, seven of the eight studies included in the meta-analysis were rated as having a high risk of bias, with Domain 4 (bias in the measurement of the outcome) the domain most likely to be rated high risk across studies.

With side effects specified as the outcome of interest, the risk of bias assessment found that of eight studies included in the meta-analysis, seven were rated as having a high risk of bias [3, 4, 36, 38, 40–42], and one was rated as having some concerns [6] (see Fig. 3 and Fig. S2). The Cohen’s Kappa statistic for overall bias was 1.00 (i.e., excellent agreement between reviewers).

### Certainty of evidence

Table S5 presents results of the GRADE assessment. For Phase 2 studies, all primary outcomes were rated as having very low certainty of evidence. For Phase 3 studies, two primary outcomes were rated as having low certainty of evidence, and one rated as moderate. For all studies, withdrawal for any reason was rated as having moderate certainty of evidence.

### Adherence to CONSORT Harms 2022

No included RCT had adequate adherence to the CONSORT Harms 2022 recommendations (>70%; Table 2). The median adherence rate was 50% (range 21–64%).

**Table 2:** Adherence of papers to CONSORT Harms 2022 checklist by study and item. 1 = adequately reported, 0 = inadequately or not reported at all.

CONSORT item			Study							Adherence of trials, n (%)
		Bouso 2011	Danforth 2018	Mitchell 2021	Mitchell 2023	Mithoefer 2018	Mithoefer 2011	Oehen 2013	Ot’alora 2018	
Title and abstract	Item 1b: Structured summary of trial design, methods, results of outcomes of benefits and harms, and conclusions (for specific guidance see CONSORT for abstracts).	1	0	1	1	1	1	1	1	7/8 (88%)
Introduction	Item 2b: Specific objectives or hypotheses for outcomes of benefits and harms.	1	1	1	0	1	1	1	0	6/8 (75%)
Methods	Item 6a: Completely defined prespecified primary and secondary outcomes, for both benefits and harms, including how and when they were assessed.	1	0	0	1	0	0	0	1	3/8 (38%)
	Item 6b: Any changes to trial outcomes after the trial commenced, with reasons.	1	n/a	n/a	n/a	n/a	n/a	n/a	n/a	1/1 (100%)
	Item 6c: Describe if and how non-prespecified outcomes of benefits and harms were identified, including any selection criteria, if applicable.	n/a	n/a	n/a	n/a	n/a	n/a	n/a	n/a	n/a
	Item 11a: If done, who was blinded after assignment to interventions (eg, participants, care providers, those assessing outcomes of benefits and harms) and how.	1	1	1	1	1	1	0	1	7/8 (88%)
	Item 12a: Statistical methods used to compare groups for primary and secondary outcomes of both benefits and harms.	0	1	0	1	0	0	0	0	2/8 (25%)
Results	Item 13a: For each group, the numbers of participants who were randomly assigned, received intended treatment, and were analyzed for outcomes of benefits and harms.	0	1	1	1	1	1	0	1	6/8 (75%)
	Item 14a: Dates defining the periods of recruitment and follow-up for outcomes of benefits and harms.	1	1	1	1	1	0	0	1	6/8 (75%)
	Item 16: For each group, number of participants (denominator) included in each analysis of outcomes of benefits and harms and whether the analysis was by original assigned groups and if any exclusions were made.	0	1	1	1	1	1	0	1	6/8 (75%)
	Item 17a: For each primary and secondary outcomes of benefits and harms, results for each group, and the estimated effect size and its 0precision (such as 95% confidence intervals).	0	0	0	0	0	0	0	0	0/8 (0%)
	It0em 17a2: For outcomes omitted from the trial report (benefits and harms), provide rationale for not reporting and indicate where the data on omitted outcomes can be accessed.	1	0	0	0	0	0	0	0	1/8 (13%)
	Item 17b: Presentation of both absolute and relative effect sizes is recommended for outcomes of benefits and harms.	0	0	0	0	0	0	0	0	0/8 (0%)
	Item 17c: Report zero events if no harms were observed.	0	1	0	0	0	0	0	0	1/8 (13%)
	Item 18: Results of any other analyses performed for outcomes of benefits and harms, including subgroup analyses and adjusted analyses, distinguishing prespecified from exploratory.	n/a	n/a	n/a	n/a	n/a	n/a	n/a	n/a	n/a
Discussion	Item 20: Trial limitations, addressing sources of potential bias related to the approach to collecting or reporting data on harms, imprecision, and, if relevant, multiplicity or selection of analyses.	0	0	1	1	0	0	0	0	2/8 (25%)
Other information	Item 24: Where the full trial protocol and other relevant documents can be accessed, including additional data on harms.	0	1	1	1	1	1	1	1	7/8 (88%)
Total		7	8	8	9	7	6	3	7	
Adherence (%)		47	57	57	64	50	43	21	50	

*CONSORT* consolidated standards of reporting trials.

### Comparison of adverse events published versus reported on ClinicalTrial.gov

We were only able to complete this analysis for five studies for non-serious adverse events and eight for serious adverse events, because there were differences in how adverse events were reported between publications and ClinicalTrial.gov, and some studies did not have an entry or data on ClinicalTrial.gov (the two studies without ClinicalTrial.gov entries were also not registered on EudraCT). In ClinicalTrial.gov registers, we found 1487 non-serious adverse events recorded, versus 661 reported in published articles (Table S6). In ClinicalTrial.gov registers, we found 13 serious adverse events recorded, versus 9 reported in published articles (Table S7). The four serious adverse events not reported in published articles all concerned participants from MDMA-AP groups and included: (1) metastases to central nervous system; (2) clavicle fracture; (3) syncope; and (4) suicidal behavior.

## Discussion

To our knowledge, this is the first comprehensive systematic review and meta-analysis of side effects of MDMA-AP across psychiatric indications. In Phase 2 studies, although participants undergoing MDMA-AP had higher odds of experiencing side effects during and 7 days after medication sessions relative to placebo-AP participants, there was no difference in TEAEs. Moreover, the observed differences between active and placebo conditions were relatively modest. For Phase 3 studies, participants undergoing MDMA-AP had higher odds of experiencing any TEAE as well as thirteen different types of TEAEs compared with placebo. These were largely transient and mild to moderate in severity. These findings should, however, be interpreted with caution; we identified marked shortcomings in published information on side effects, including heterogeneity and weaknesses in how side effects were defined, assessed, and reported. Indeed, the certainty of evidence was rated as very low or low for 6 of 8 primary outcomes, with 2 rated as moderate certainty. This indicates that much remains unknown about the safety profile of MDMA-AP.

In Phase 2 studies, MDMA-AP participants had 1.7 times greater odds of experiencing any side effect during medication sessions than placebo participants, and 1.6 greater odds of side effects in the 7 days following. These results, however, were based on ‘spontaneously reported reactions’ data, defined as a subset of adverse events that could be expected based on findings in healthy volunteers [28]. It is therefore unsurprising that an effect was detected, albeit a small one [49]. Participants treated with MDMA-AP also had 4.7 greater odds of anxiety and 4.8 greater odds of jaw clenching during medication sessions relative to placebo participants. Anxiety was also prominent in PTSD patients, who had 4.1 greater odds of anxiety during medication sessions when treated with MDMA-AP, and 4.8 greater odds in the 7 days following compared to the placebo-AP group. These findings are consistent with results of previous meta-analyses [14, 23, 24] and evidence from healthy subjects [11, 50]. No differences between the MDMA-AP and control groups were observed in the odds of experiencing any TEAE, psychiatric TEAE or in withdrawal from the study, suggesting that side effects typically resolved within 7 days of MDMA-AP sessions and did not result in study withdrawal. Contrary to expectations, sub-group analyses found that people with PTSD who received MDMA-AP had 0.5 lower odds of experiencing any TEAE. Notably, only two studies were included in this meta-analysis, both of which used a low-dose MDMA active control rather than inert placebo. While active placebos improve blinding in studies evaluating MDMA-AP, this may complicate comparison of safety data between groups where treatments are not pharmacologically-distinct [1].

In Phase 3 studies, MDMA-AP participants had 3.5 times greater odds of experiencing any TEAE compared with placebo participants. They also had increased odds of muscle tightness, decreased appetite, nausea, excessive perspiration, feeling cold, restlessness, dilated pupils, jaw clenching, uncontrolled eye movements, feeling jittery, non-cardiac chest pain/discomfort, blurred vision, and chills. The higher number of adverse events reaching significance in Phase 3 studies was likely due to increased statistical power, given the larger sample size (*N* = 194). These results are largely consistent with evidence about the acute and residual effects of MDMA in healthy subjects [50], suggesting that the side effects of MDMA-AP are primarily due to direct effects of MDMA (or that side effects arising from the combination of MDMA and psychotherapy are not well detected using the methods employed). No differences between MDMA-AP and control groups were observed in the odds of experiencing AESIs related to suicide risk or cardiac function, however, these are likely rare adverse events that were unlikely to reach significance in the meta-analysis. Nonetheless, AESIs related to suicidality were reported at rates which were similar or lower in the MDMA group compared with control. Regardless of assignment, incidences of suicidality should be an important safety consideration in MDMA-AP trials, as there may be specific risks for people randomized to placebo, given the possibility of high expectations driven by hype about psychedelics coupled with blinding failure [1, 2].

While all RCTs included aimed to examine MDMA-AP’s safety, none had adequate adherence to the CONSORT Harms 2022 recommendations for reporting of safety data. Reporting rates for adverse events were also low: 56% of non-serious and 31% of serious adverse events recorded on ClinicalTrial.gov registers were not reported in published articles. The discrepancy in non-serious adverse events was primarily driven by the two Phase 3 studies, which only reported adverse events with at least a two-fold difference between the MDMA-AP and placebo-AP groups. In addition, two studies [36, 38] stated that there were ‘no drug-related serious adverse events’, but otherwise did not systematically report on adverse events. Among the four serious adverse events not reported in these papers, there was one instance of syncope and one of suicidal behavior in the MDMA-AP groups. Greater transparency is required regarding the timing of these events (i.e., did they occur during or immediately after medication sessions?) and the rationale for deeming them unrelated to condition.

This review identified several limitations of existing MDMA-AP evidence. First, although most studies reported side effect information, reporting practices were largely insufficient. This finding is not limited to MDMA-AP research (e.g., see [29, 51]) and is consistent with concerns about safety reporting in trials of mental health interventions more broadly [52]. Indeed, evidence from clinical trials outside of psychiatry also suggest that compliance with the guidelines on reporting of harms is often inadequate (e.g., [53, 54]). Greater emphasis on the CONSORT Harms 2022 recommendations during review is recommended to improve reporting of side effect information. Heterogeneous reporting practices across studies also placed limitations on our ability to combine side effect data for meta-analyses. Most prominently, we had to analyze data from Phase 2 and Phase 3 studies separately due to differences in assessment and reporting of side effects. Further, we could only draw conclusions regarding acute and short-term side effects because insufficient data were available regarding longer-term risks beyond active treatment.

Second, certainty of the evidence was rated as very low for Phase 2 side effect outcomes, and low to moderate for Phase 3. The improvement in certainty of the evidence in Phase 3 studies was mainly attributable to their larger more ethno-racially diverse samples. The overall evidence on MDMA-AP, however, consists of a relatively limited number of studies with highly selective samples (i.e., most people volunteering for these studies were excluded from participation). While appropriate for this early stage of research, this means we cannot yet with high confidence determine the safety profile or risk-benefit ratio of MDMA-AP. It is likely that implementation beyond controlled clinical trials – in people with more complex needs—will increase the incidence of side effects. In addition, while heterogeneity was low, in many cases estimates lacked precision. We also could not formally test for publication bias, due to the low number of studies available (<10), however possible sources of publication bias were identified, including the body of evidence largely consisting of small studies sponsored by a single advocacy group [34]. Taken together, there is a need for further independent clinical trials, with larger more representative samples to enable more definitive conclusions about the safety of MDMA-AP.

Third, consistent with a previous review [14], we found that most studies relied on passive monitoring of spontaneously reported side effects. While spontaneous reporting avoids the problem of suggestive questions influencing outcomes, evidence suggests that it likely underestimates the extent of side effects compared with a more systematic approach [55]. One implication is that alongside spontaneous reporting, studies of MDMA-AP should also use systematic checklists or scales to assess expected and general side effects which appear acutely, between doses, and at long-term follow-up. This is particularly important at this early stage of research as the data build about the risk-benefit profile of MDMA-AP [55].

Fourth, given differences in dosage, sample sizes, and reporting procedures between Phase 2 and 3 studies, we were unable to assess whether side effects differed as a function of trial design e.g., for instance due to nocebo effects in placebo-controlled studies. Future trials using comparative effectiveness designs could provide further information about the extent to which expectations contribute to the effects (therapeutic or adverse) of MDMA-AP versus control conditions (e.g., see [56]). Lastly, only four studies assessed the dose-dependent nature of side effects. It is critical for future studies to examine at what dose each side effect emerges to identify the ideal dosage and reduce unnecessary risks.

## Conclusion

The evidence synthesized indicates that relative to placebo-AP, MDMA-AP is associated with greater likelihood of experiencing mild to moderate, but largely transient side effects. Findings, however, need to be interpreted cautiously due to limitations of the existing evidence. Our review revealed a need to improve practices for assessing side effects and adherence to reporting guidelines for harms in MDMA-AP studies. Based on results reported to date, any assessment of the risk-benefit profile of MDMA-AP remains limited due to the identified issues. Further large-scale trials which systematically assess side effects (including long-term follow up), comprehensively report all potential side-effects and follow CONSORT Harms guidelines are recommended. In Australia, and other countries soon expected to implement MDMA-AP, systematic, independent post-marketing studies of real-world side effects are also needed.

### Supplementary information

Supplementary Material

Supplementary Table 3

Supplementary Table 8

## Data Availability

Data are available upon request from the authors.
