# Supplementary material for: Side-effects of mdma-assisted psychotherapy: a systematic review and meta-analysis
Source: Neuropsychopharmacology. 2024 Apr 23;49(8):1208–26. doi: 10.1038/s41386-024-01865-8 (PMC11224236; doi:10.1038/s41386-024-01865-8)
Supplement: Supplementary file 1 — Supplementary Material [file 41386_2024_1865_MOESM1_ESM.docx]

**Supplementary Table 1. Search Strategy**

| **Pubmed** | (((MDMA*[Title/Abstract] OR 3,4-methylenedioxymethamphetamine*[Title/Abstract]) AND (psychotherapy[Title/Abstract] OR therapy[Title/Abstract])) AND (safe*[Title/Abstract] OR side*[Title/Abstract] OR adverse*[Title/Abstract])) OR ((MDMA*[Other Term] OR 3,4- methylenedioxymethamphetamine*[Other Term]) AND (psychotherapy[Other Term] OR therapy[Other Term]) AND (safe*[Other Term] OR side*[Other Term] OR adverse*[Other Term])) |
| --- | --- |
| **EMBASE (Ovid)** | ((MDMA* or 3,4-methylenedioxymethamphetamine*) and (psychotherapy or therapy) and (safe* or side* or adverse*)).ti. OR ((MDMA* or 3,4-methylenedioxymethamphetamine*) and (psychotherapy or therapy) and (safe* or side* or adverse*)).ab. OR ((MDMA* or 3,4-methylenedioxymethamphetamine*) and (psychotherapy or therapy) and (safe* or side* or adverse*)).kw. |
| **PsycINFO (Ovid)** | ((MDMA* or 3,4-methylenedioxymethamphetamine*) and (psychotherapy or therapy) and (safe* or side* or adverse*)).ti. OR ((MDMA* or 3,4-methylenedioxymethamphetamine*) and (psychotherapy or therapy) and (safe* or side* or adverse*)).ab. OR ((MDMA* or 3,4-methylenedioxymethamphetamine*) and (psychotherapy or therapy) and (safe* or side* or adverse*)).id. |
| **MEDLINE (Ovid)** | ((MDMA* or 3,4-methylenedioxymethamphetamine*) and (psychotherapy or therapy) and (safe* or side* or adverse*)).ti. OR ((MDMA* or 3,4-methylenedioxymethamphetamine*) and (psychotherapy or therapy) and (safe* or side* or adverse*)).ab. OR ((MDMA* or 3,4-methylenedioxymethamphetamine*) and (psychotherapy or therapy) and (safe* or side* or adverse*)).kw. |
| **Cochrane Central Register of Controlled Trials (CENTRAL)** | (MDMA* OR 3,4-methylenedioxymethamphetamine*):ti,ab,kw AND (psychotherapy OR therapy):ti,ab,kw AND (safe* OR side* OR adverse):ti,ab,kw |

**Supplementary Table 2. Studies which did not contribute data to the meta-analyses or calculation of summary event rates**

| **Study** | **Study population** | **Sample size** | **Reason not included** |
| --- | --- | --- | --- |
| **Jardim et al, 2021** | PTSD | 3 | No control group - Open-label |
| **Jerome et al, 2020** | PTSD | 107* | No control group - LTFU, pooled analysis of 6 Phase 2 trials |
| **Monson et al, 2020** | Couples, with one member diagnosed with PTSD | 12 (six couples) | No control group - Open-label |
| **Oehen & Gasser 2022** | Various treatment-resistant disorders | n = 50 MDMA only (n = 6) MDMA and LSD (n = 18) | No control group - Case-series |
| **Sessa et al, 2021** | Alcohol Use Disorder | 14 | No control group - Open-label |

*8 participants did not complete treatment, and six of the eight participants underwent at least one medication session prior to discontinuing study participation.

LTFU: long-term follow-up; PTSD: Post traumatic stress disorder

**
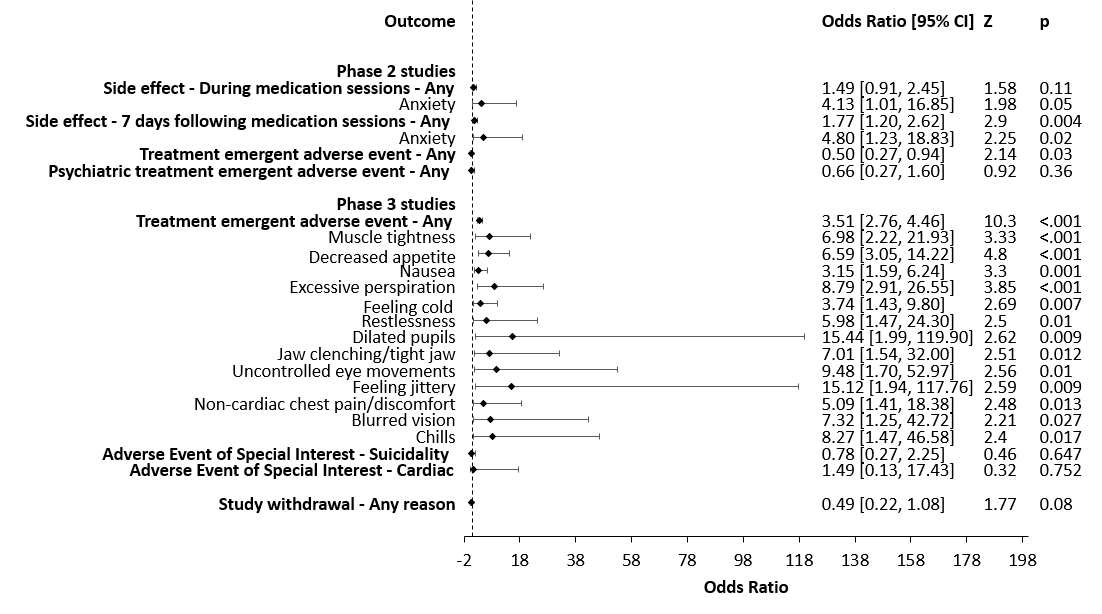
**

**Supplementary Figure 1. Summary of meta-analysis results – PTSD studies only:** Odds ratios (OR) and 95% confidence intervals (CIs) comparing MDMA-AP with control on all primary outcomes and secondary outcomes that achieved statistical significance (p < 0.05). OR > 1 indicates an increased likelihood of the event when treated with MDMA-AP compared with control and an OR < 1 indicates a reduced likelihood.

**Supplementary Table 4. Studies included/excluded from particular analyses**

| **Study** | **Study population** | **Sample size** | **Included in meta-analysis** | **Excluded from meta-analysis** |
| --- | --- | --- | --- | --- |
| **Bouso 2011**  **Phase 2** | PTSD | 6 | Side effects - During 7 days following medication sessions (extracted from UKU Scale of Secondary Effects data) | No systematic recording of:   - Side effects - During medication sessions - TEAEs - Psychiatric TEAEs - Withdrawal |
| **Danforth 2018**  **Phase 2** | Autistic adults with social anxiety | 12 | - Side effects - During medication sessions - Side effects - 7 days following medication sessions - TEAEs, by SOC - Psychiatric TEAEs - Withdrawal | n/a |
| **Mitchell 2021**  **Phase 3** | PTSD | 90 | - TEAEs, by type - AESIs - Withdrawal | n/a |
| **Mitchell 2023**  **Phase 3** | PTSD | 104 | - TEAEs, by type - AESIs - Withdrawal | n/a |
| **Mithoefer 2018**  **Phase 2** | PTSD | 26 | - Side effects - During medication sessions - Side effects - 7 days following medication sessions - TEAEs, by SOC - Psychiatric TEAEs - Withdrawal | Open-label data |
| **Mithoefer 2011**  **Phase 2** | PTSD | 20 | - Withdrawal | Open-label data  Side effects during and 7 days following medication sessions reported by number of events, not number of participants so odds ratios could not be calculated.  No systematic recording of:   - TEAEs - Psychiatric TEAEs |
| **Oehen 2013**  **Phase 2** | PTSD | 12 | - Withdrawal | Open-label data  Side effects during and 7 days following medication sessions reported by number of events, not number of participants so odds ratios could not be calculated. |
| **Ot’alora 2018**  **Phase 2** | PTSD | 28 | - Side effects - During medication sessions - Side effects - 7 days following medication sessions - TEAEs, by SOC - Psychiatric TEAEs - Withdrawal | Open-label data |

SOC: System Organ Class; TEAE: Treatment Emergent Adverse Event; AESI: Adverse Event of Special Interest

**
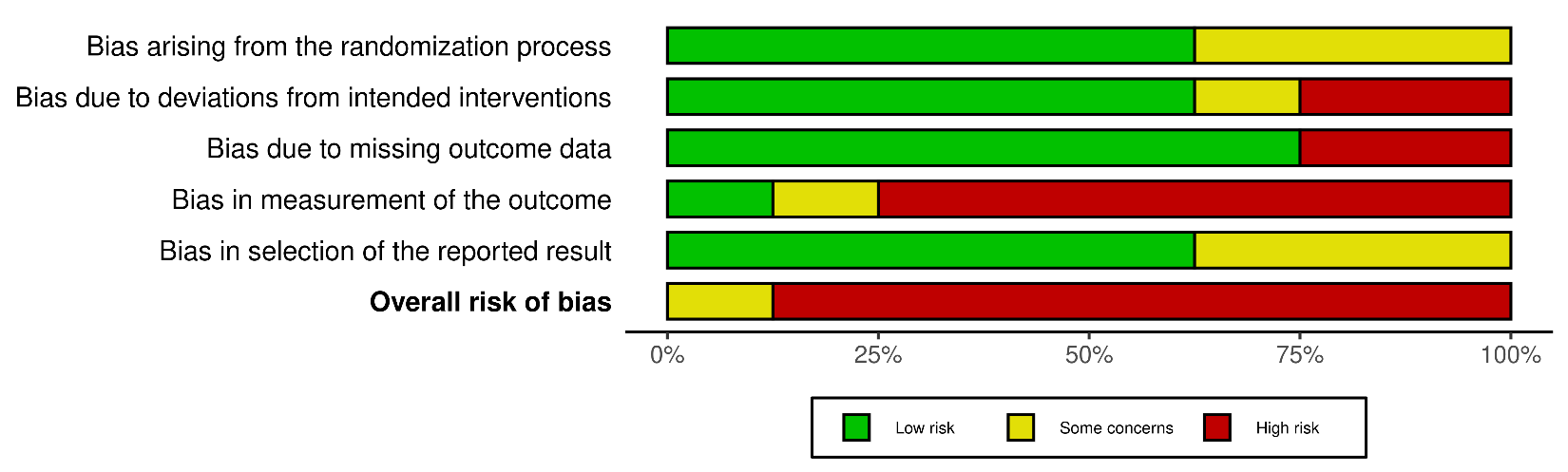
**

Supplementary Figure 2. Summary of quality assessment of studies included in the meta-analysis

**Supplementary Table 5. GRADE Summary of Findings Table**

| **Outcome** | **Total sample size (no. of studies)** | **Risk of Bias^1^** | **Inconsistency^2^** | **Indirectness^3^** | **Imprecision^4^** | **Publication Bias^5^** | **Certainty** |
| --- | --- | --- | --- | --- | --- | --- | --- |
| **Phase 2 studies** | | | | | | | |
| Side effect – During medication sessions - Any side effect | 66 (3) | Serious limitations | No evidence of significant heterogeneity | Serious limitations | Serious limitations | Moderate | Very low |
| Side effect – 7 days following medication sessions - Any side effect | 72 (4) | Serious limitations | No evidence of significant heterogeneity | Serious limitations | Serious limitations | Moderate | Very low |
| Treatment emergent adverse event - Any event | 66 (3) | Serious limitations | No evidence of significant heterogeneity | Serious limitations | Serious limitations | Moderate | Very low |
| Psychiatric treatment emergent adverse event - Any event | 66 (3) | Serious limitations | No evidence of significant heterogeneity | Serious limitations | Serious limitations | Moderate | Very low |
| **Phase 3 studies** | | | | | | | |
| Treatment emergent adverse event - Any event | 194 (2) | Serious limitations | No evidence of significant heterogeneity | No serious limitations | No serious limitations | Moderate | Moderate |
| Adverse Event of Special Interest – Suicidality | 194 (2) | Serious limitations | No evidence of significant heterogeneity | No serious limitations | Serious limitations | Moderate | Low |
| Adverse Event of Special Interest – Cardiac | 194 (2) | Serious limitations | No evidence of significant heterogeneity | No serious limitations | Serious limitations | Moderate | Low |
| **All studies** | | | | | | | |
| Withdrawal - Any reason | 280 (6) | Serious limitations | No evidence of significant heterogeneity | Some limitations | Some limitations | Moderate | Moderate |

^1^Downgraded one level due to high risk of bias in majority of studies

^2^Derived from I^2^ statistics (Supplementary Table 3)

^3^Outcomes with serious limitations downgraded one level because the majority of data came from studies which used selective, non-representative samples

^4^Outcomes with serious limitations downgraded one level because data came from small studies; 95% CI’s were sufficiently wide to include both a meaningful result in one direction and negligible effect; or there were few observed events

^5^It was not possible to test for publication bias using funnel plots, so this criteria was assessed in terms of the number and size of studies and possible conflicts of interest in study sponsors

**Supplementary Table 6.** Summary of adverse events reported in published articles and ClinicalTrial.gov Register

| **Study** | **Phase** | **Threshold for reporting adverse events** | | **Total (non-serious) adverse events** | |
| --- | --- | --- | --- | --- | --- |
|  |  | **PA** | **CTR** | **PA** | **CTR** |
| Mitchell 2021 | Blinded treatment period | 5%^1^ | 5% | 210^1^ | 638 |
| Mitchell 2023 | Blinded treatment period | 0-10%^2^ | 5% | 413^2^ | 648 |
| Monson 2020 | Treatment period | nr | 5% | 33 | 34 |
|  | 6-month follow-up |  |  | 5 | 5 |
| Mithoefer 2011 | Blinded treatment period | nr | 5% | nr | 91 |
|  | Open-label | nr | 5% | nr | 21 |
| Oehen 2013 | Blinded treatment period | nr | 5% | nr | 41 |
|  | Open-label | nr | 5% | nr | 9 |

nr: Not reported; PA: Published article; CTR: ClinicalTrial.gov register

^1^Reported the most common (>5% of subjects) adverse events with at least twice the prevalence in the MDMA group versus the placebo.

^2^Reported psychiatric adverse events that occurred in >5% of participants in either group. Reported all cardiac and vascular adverse events. Otherwise, reported the most common (>10% subjects) adverse events with at least twice the prevalence in the MDMA group versus the placebo.

**Supplementary Table 7.** Summary of serious adverse events reported in published articles and ClinicalTrial.gov Register

| **Study** | **Phase** | **Total serious adverse events** | |
| --- | --- | --- | --- |
|  |  | **PA** | **CTR** |
| Mitchell 2021 | Blinded treatment period | 2 | 2 |
| Mitchell 2023 | Blinded treatment period | 0 | 0 |
| Monson 2020 | Treatment period | 0 | 0 |
|  | 6-month follow-up | 0 | 0 |
| Danforth 2018 | Blinded treatment period | 0 | 0 |
|  | Open-label | nr | 0 |
| Mithoefer 2018 | Blinded treatment period | 4 | 4 |
|  | Open-label |  |  |
| Ot’alora 2018 | Blinded treatment period | 0 | 1 |
|  | Open-label | 1 | 0 |
|  | 12-month follow up | 2 | 2 |
| Mithoefer 2011 | Blinded treatment period | nr^1^ | 2 |
|  | Open-label | nr^1^ | 0 |
| Oehen 2013 | Blinded treatment period | nr^1^ | 2 |
|  | Open-label | nr^1^ | 0 |

nr: Not reported; PA: Published article; CTR: ClinicalTrial.gov register

^1^Reported that there were no drug-related serious adverse events
